# Supplementary material for: Saccharomyces boulardii (CNCM I-745) improves intestinal damage in sepsis by remodeling intestinal flora structure
Source: Front Cell Infect Microbiol. 2025 Jul 31;15:1602792. doi: 10.3389/fcimb.2025.1602792 (PMC12350329; doi:10.3389/fcimb.2025.1602792)
Supplement: Supplementary file 1 [file DataSheet1.zip › summary/summary/7_Differential_analysis/2_LEfSe_result/Group_DW4_vs_DW0_vs_CW4_vs_CW0_vs_BW4_vs_BW0_vs_AW4_vs_AW0_0.05_3.0/LEfSe_Group_DW4_vs_DW0_vs_CW4_vs_CW0_vs_BW4_vs_BW0_vs_AW4_vs_AW0.cladogram.pdf]

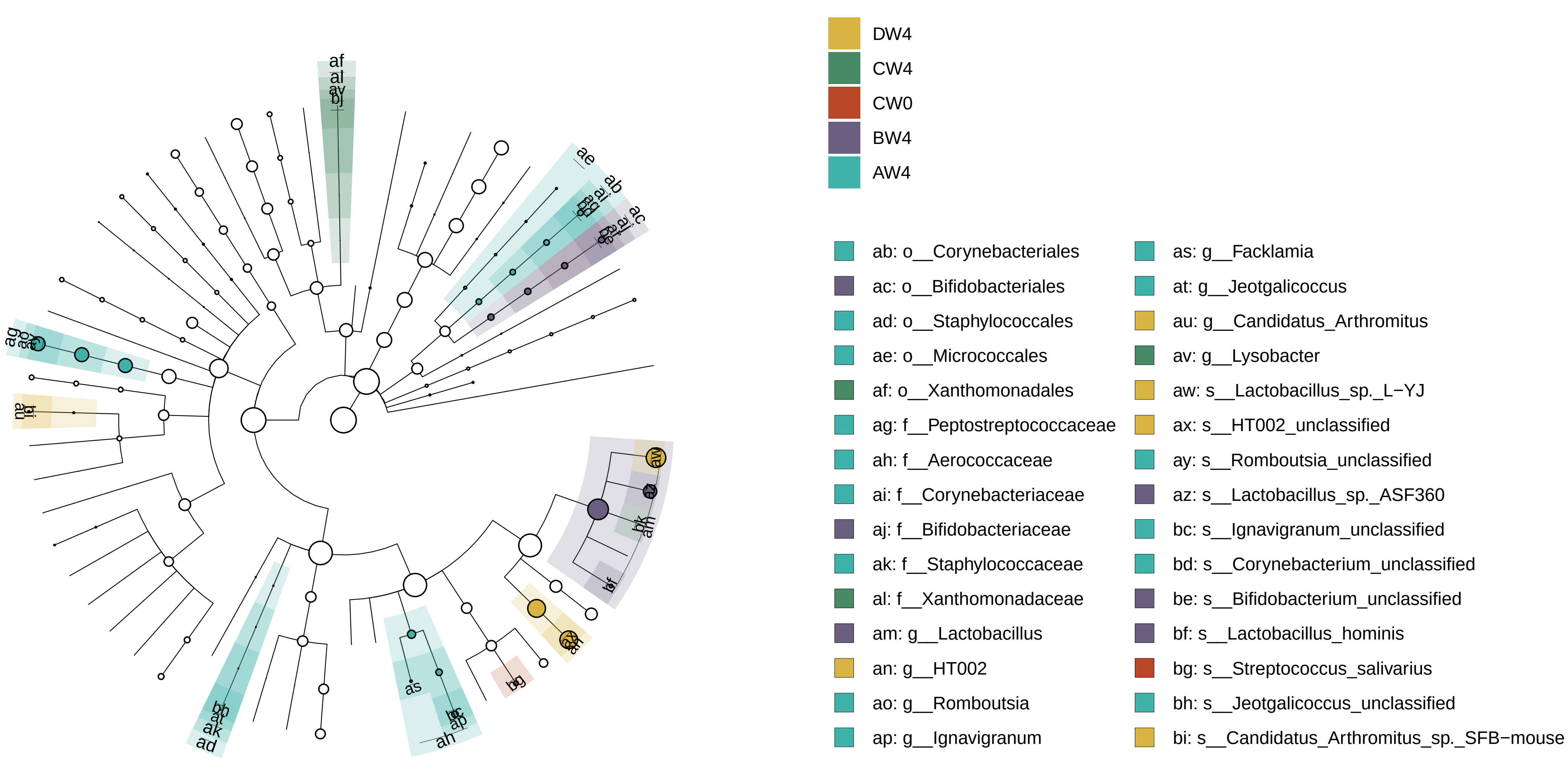
DW4
CW4
CW0
BW4
AW4
ab: o__Corynebacteriales
ac: o__Bifidobacteriales
ad: o__Staphylococcales
ae: o__Micrococcales
af: o__Xanthomonadales
ag: f__Peptostreptococcaceae
ah: f__Aerococcaceae
ai: f__Corynebacteriaceae
aj: f__Bifidobacteriaceae
ak: f__Staphylococcaceae
al: f__Xanthomonadaceae
am: g__Lactobacillus
an: g__HT002
ao: g__Romboutsia
ap: g__Ignavigranum
aq: g__Corynebacterium
ar: g__Bifidobacterium
as: g__Facklamia
at: g__Jeotgalicoccus
au: g__Candidatus_Arthromitus
av: g__Lysobacter
aw: s__Lactobacillus_sp._L−YJ
ax: s__HT002_unclassified
ay: s__Romboutsia_unclassified
az: s__Lactobacillus_sp._ASF360
bc: s__Ignavigranum_unclassified
bd: s__Corynebacterium_unclassified
be: s__Bifidobacterium_unclassified
bf: s__Lactobacillus_hominis
bg: s__Streptococcus_salivarius
bh: s__Jeotgalicoccus_unclassified
bi: s__Candidatus_Arthromitus_sp._SFB−mouse
bj: s__Lysobacter_defluvii
bk: s__Lactobacillus_intestinalis
